# Dual SGLT-1 and SGLT-2 inhibition improves left atrial dysfunction in HFpEF

**DOI:** 10.1186/s12933-020-01208-z

**Published:** 2021-01-07

**Authors:** David Bode, Lukas Semmler, Paulina Wakula, Niklas Hegemann, Uwe Primessnig, Nicola Beindorff, David Powell, Raphael Dahmen, Hartmut Ruetten, Christian Oeing, Alessio Alogna, Daniel Messroghli, Burkert M. Pieske, Frank R. Heinzel, Felix Hohendanner

**Affiliations:** 1 Department of Internal Medicine and Cardiology, Charité University Medicine, Campus Virchow-Klinikum, Augustenburgerplatz 1, 13353 Berlin, Germany; 2 German Center for Cardiovascular Research (DZHK), Partner Site Berlin, Berlin, Germany; 3 Lexicon Pharmaceuticals, Metabolism Research, Houston, TX USA; 4 Sanofi-Aventis Deutschland GmbH, Research & Development, 65926 Frankfurt am Main, Germany; 5 Department of Internal Medicine and Cardiology, German Heart Center Berlin, 13353 Berlin, Germany; 6 Berlin Institute of Health (BIH), Berlin, Germany; 7 Berlin Experimental Radionuclide Imaging Center (BERIC), Charité-Universitaetsmedizin Berlin, Berlin, Germany

**Keywords:** Atrial cardiomyopathy, Heart failure with preserved ejection fraction, SGLT inhibition, Atrial remodeling, Left atrial cardiomyocytes, Calcium cycling, Mitochondria

## Abstract

**Background:**

Sodium–glucose linked transporter type 2 (SGLT-2) inhibition has been shown to reduce cardiovascular mortality in heart failure independently of glycemic control and prevents the onset of atrial arrhythmias, a common co-morbidity in heart failure with preserved ejection fraction (HFpEF). The mechanism behind these effects is not fully understood, and it remains unclear if they could be further enhanced by additional SGLT-1 inhibition. We investigated the effects of chronic treatment with the dual SGLT-1&2 inhibitor sotagliflozin on left atrial (LA) remodeling and cellular arrhythmogenesis (i.e. atrial cardiomyopathy) in a metabolic syndrome-related rat model of HFpEF.

**Methods:**

17 week-old ZSF-1 obese rats, a metabolic syndrome-related model of HFpEF, and wild type rats (Wistar Kyoto), were fed 30 mg/kg/d sotagliflozin for 6 weeks. At 23 weeks, LA were imaged in-vivo by echocardiography. In-vitro, Ca^2+^ transients (CaT; electrically stimulated, caffeine-induced) and spontaneous Ca^2+^ release were recorded by ratiometric microscopy using Ca^2+^-sensitive fluorescent dyes (Fura-2) during various experimental protocols. Mitochondrial structure (dye: Mitotracker), Ca^2+^ buffer capacity (dye: Rhod-2), mitochondrial depolarization (dye: TMRE) and production of reactive oxygen species (dye: H2DCF) were visualized by confocal microscopy. Statistical analysis was performed with 2-way analysis of variance followed by post-hoc Bonferroni and student’s t-test, as applicable.

**Results:**

Sotagliflozin ameliorated LA enlargement in HFpEF in-vivo. In-vitro*,* LA cardiomyocytes in HFpEF showed an increased incidence and amplitude of arrhythmic spontaneous Ca^2+^ release events (SCaEs). Sotagliflozin significantly reduced the magnitude of SCaEs, while their frequency was unaffected. Sotagliflozin lowered diastolic [Ca^2+^] of CaT at baseline and in response to glucose influx, possibly related to a ~ 50% increase of sodium sodium–calcium exchanger (NCX) forward-mode activity. Sotagliflozin prevented mitochondrial swelling and enhanced mitochondrial Ca^2+^ buffer capacity in HFpEF. Sotagliflozin improved mitochondrial fission and reactive oxygen species (ROS) production during glucose starvation and averted Ca^2+^ accumulation upon glycolytic inhibition.

**Conclusion:**

The SGLT-1&2 inhibitor sotagliflozin ameliorated LA remodeling in metabolic HFpEF. It also improved distinct features of Ca^2+^-mediated cellular arrhythmogenesis in-vitro (i.e. magnitude of SCaEs, mitochondrial Ca^2+^ buffer capacity, diastolic Ca^2+^ accumulation, NCX activity). The safety and efficacy of combined SGLT-1&2 inhibition for the treatment and/or prevention of atrial cardiomyopathy associated arrhythmias should be further evaluated in clinical trials.

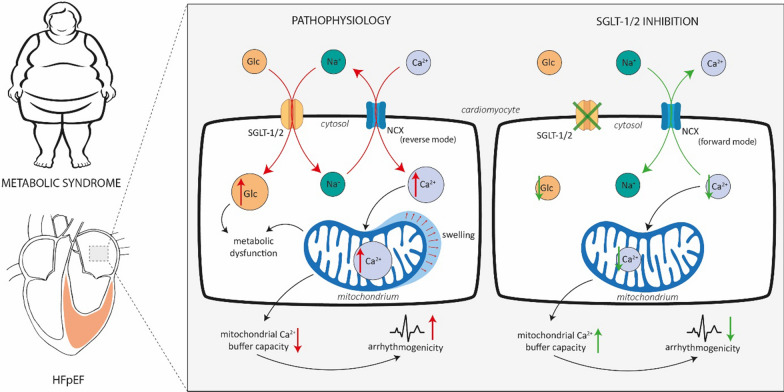

## Background

Heart failure (HF) with preserved ejection fraction (HFpEF) is an increasingly prevalent disease. Left atrial (LA) cardiomyopathy and remodeling are hallmark features of HFpEF and commonly associated with LA enlargement and (precursors of) atrial fibrillation (AF) [1–3]. Catheter ablation, rather than medical therapy (rate/rhythm control), is currently the most effective treatment for AF to reduce mortality and heart failure hospitalization in patients with HF with reduced ejection fraction (HFrEF) [4]. Investigators of the DECLARE-TIMI 58 trial have recently reported that the sodium glucose-linked transporter (SGLT) inhibitor dapagliflozin reduces the risk of AF events by 19% in patients with type 2 diabetes mellitus (T2DM) with cardiovascular risk factors, regardless of the patients’ previous history of AF [5]. In the DAPA-HF trial, dapagliflozin reduced cardiovascular mortality in HFrEF, (independent of the presence of T2DM) [6] and trials investigating SGLT-2 inhibition in HFpEF are ongoing.

SGLT inhibitors were designed to block the eponymous transporter in the proximal (SGLT-2) and distal (SGLT-1) tubule, which is responsible for the reabsorption of glucose filtered by the kidney glomerulus [7]. However, there is a growing body of evidence, that SGLT inhibition exerts its cardiovascular benefits beyond glycemic control, especially in patients with diabetes and heart failure [8]. Various sites-of-action (and an interplay thereof) have been suggested, e.g. [Na^+^] of cardiomyocytes, mitochondrial dysfunction and oxidative stress, endothelial inflammation, fibrosis and cardiac bioenergetics [9].

Gliflozins show varying SGLT type 2 over type 1 selectivity (empagliflozin: 2680-fold, dapagliflozin: 1242-fold, canagliflozin: 155-fold) [7]. Sotagliflozin (Sota) was designed to exert a 20-fold lower affinity for SGLT type 2 over type 1, while the presence of SGLT-2 in the heart, particular across species, remains a question of debate [10, 11]. SGLT-1 inhibition reduces glucose uptake in the proximal intestine, which significantly blunts and delays postprandial hyperglycemia [12]. SGLT-1 is expressed in human ventricular and atrial myocardium [13] heart and is associated with elevated Na^+^ and glucose influx in cardiomyocytes of HF patients with T2DM or obesity [14]. Humans with decreased functional SGLT-1 exhibit improved survival and decreased prevalence of HF [15]. While this effect was linked to improved glucose tolerance, these outcomes could also, at least in part, be mediated by improved cardiovascular function. Overall, cardiac SGLT-1 is perturbed in various cardiovascular disease entities and could pose an interesting target beyond glycemic control [16].

We hypothesized, that chronic treatment with the dual SGLT-1&2 inhibitor Sota mitigates LA remodeling and cellular arrhythmogenesis in a rat model of metabolic syndrome-related HFpEF. Our study explores the influence of Sota on the interplay of established pro-arrhythmic entities: LA enlargement, Ca^2+^ cycling and buffer capacity, mitochondrial (dys)function and glucose metabolism.

## Methods

### Heart failure model

Animal experiments were approved by local authorities (G0317/17 and G0276/16). The ZSF-1 obese rat model is based on a leptin receptor mutation resulting in severe metabolic dysfunction [17]. The model has repeatedly been reported to show distinct features of HFpEF, such as an increased left ventricular (LV) end diastolic pressure, LV hypertrophy, diastolic dysfunction, lung congestion and LA remodeling, while maintaining a preserved ejection fraction (EF) [18–21]. Wild-type (WT) rats (Wistar Kyoto and HFpEF (ZSF-1 obese) animals were obtained at 10 weeks (Charles River Laboratories, MA, USA) and fed a high caloric diet (Purina 5008; LabDiet, MO, USA). At 16 weeks, animals were randomly assigned to receive treatment (oral feeding) with either vehicle or the dual SGLT-1&2 inhibitor Sota (30 mg/kg/day; reported to exhibit near maximal urinary glucose secretion in rats [22]) for 7 weeks until final experiments were performed.

### Serum biomarkers

Serum biomarkers were assessed by a licensed laboratory for veterinary diagnostics (Institut für veterinärmedizinische Diagnostik, Germany) using validated photometric (β-hydroxybutyrate) and enzymatic (creatinine, urea) assays.

### Echocardiography

Echocardiography was performed and analyzed as previously described [21] by an experienced observer (N.H.) blinded to the treatment group immediately prior to sacrifice using a vevo lab ultrasound system to assess LA size and LV fractional shortening in-vivo. 1-lead electrocardiograms were obtained during echocardiography and the presence or absence of atrial rhythm disorders i.e. atrial fibrillation was documented.

### Cardiomyocyte isolation

LA and LV cardiomyocytes were isolated using enzymatic digestion as previously described in detail [23].

### Solutions and chemicals

Chemicals were obtained from Sigma-Aldrich (St. Louis, MO, USA) unless noted otherwise. The dual SGLT-1&2 inhibitor sotagliflozin was provided by Lexicon Pharamceuticals (The Woodlands, TX, USA). Fluorescent dyes Fura-2 AM, Rhod-2 AM, MitoTracker red, MitoTracker green, TMRE and H2-DCF were obtained from Thermo Fisher Scientific (Waltham, MA, USA). Tyrode solution contained (in mM): 130 NaCL, 4 KCl, 2 CaCl, 1 MgCl_2_, 10 Glucose, 10 HEPES; pH adjusted to 7.4 with NaOH. LA cardiomyocytes were plated on laminin-coated glass coverslips.

**Fig. 1:**
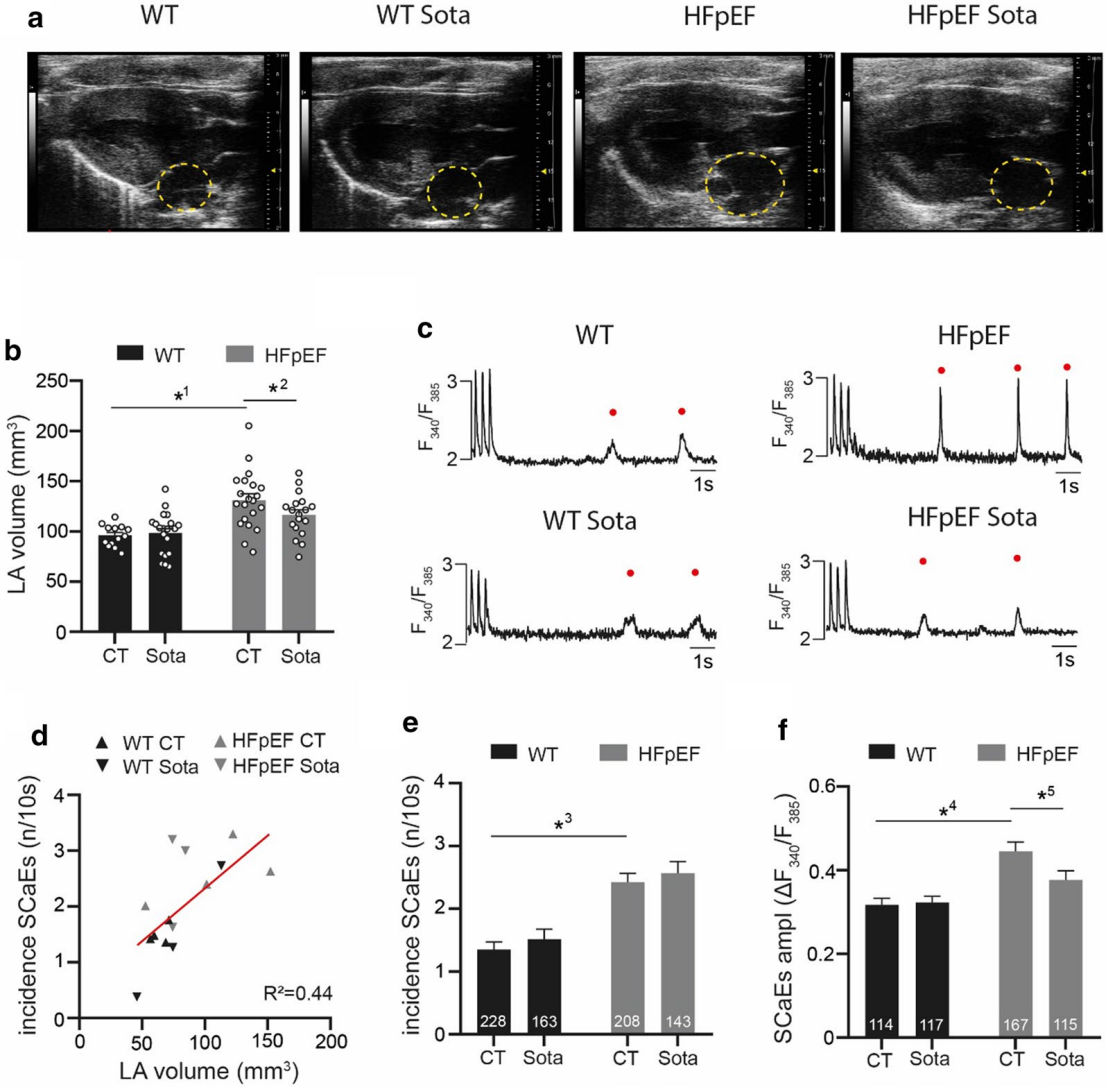
**a** Representative B-mode images in parasternal long axis view. Yellow, dashed circles indicate the LA. **b** Related data of LA volume. **c** Representative recordings of spontaneous Ca^2+^ release during a non-stimulated interval, following stimulation at 3 Hz for 10 s. **d** Linear regression of arrhythmic SR Ca^2+^ release events (SCaEs) in-vitro (average per animal) and LA volume in-vivo. **e** Occurrence of SCaEs and their **f** corresponding CaT amplitude (average per cell, respectively). Statistical analysis: Two-way ANOVA followed by post-hoc Bonferroni. *p*-values: ^1^0.001, ^2^0.045, ^3^< 0.001, ^4^0.028, ^5^< 0.001. **b** n = animals, **e**, **f** n = cells derived from 6 animals per group

**Fig. 2:**
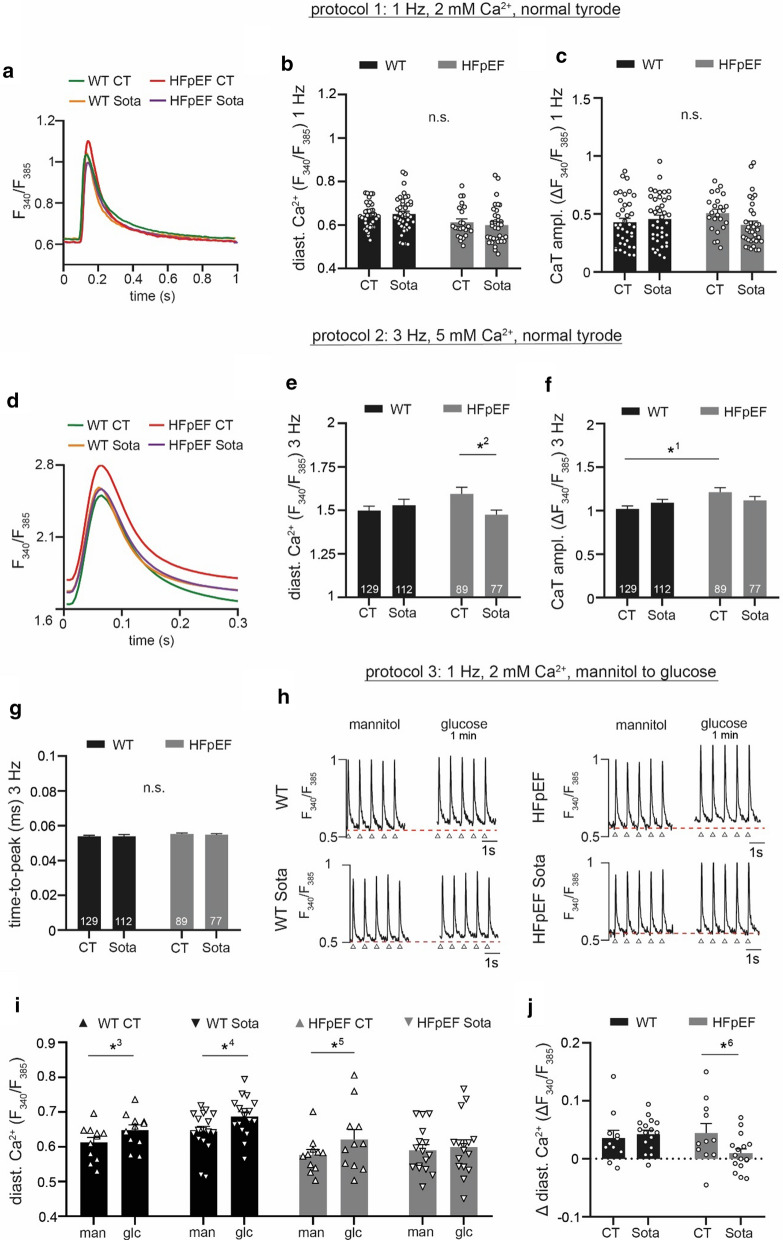
**a** Representative examples of CaT during 1 Hz electric stimulation and 2 mM extracellular [Ca^2+^]. Related data of **b** diastolic Ca^2+^ and **c** CaT amplitude. **d** Averaged CaT (all cells per group) during 3 Hz electric stimulation and 5 mM extracellular [Ca^2+^]. Related data of **e** diastolic Ca^2+^, **f** CaT amplitude and **g** time-to-peak. **h** Representative examples of CaT at 1 Hz electric stimulation after 1 h incubation in glucose-deprived buffer (left) and after reintroduction of 30 mM glucose for 1 min (right). Red, dashed lines indicate diastolic Ca^2+^ at baseline, arrows indicate stimulation triggers. **i** Related data of diastolic Ca^2+^ before (man) and after treatment with glucose (glc); **j** change of diastolic Ca^2+^. Statistical analysis: two-way ANOVA followed by post-hoc Bonferroni. *p*-values: ^1^0.03, ^2^0.02, ^3^0.01, ^4^< 0.001, ^5^0.001, ^6^0.046. n = cells derived from 6 animals per group

**Fig. 3:**
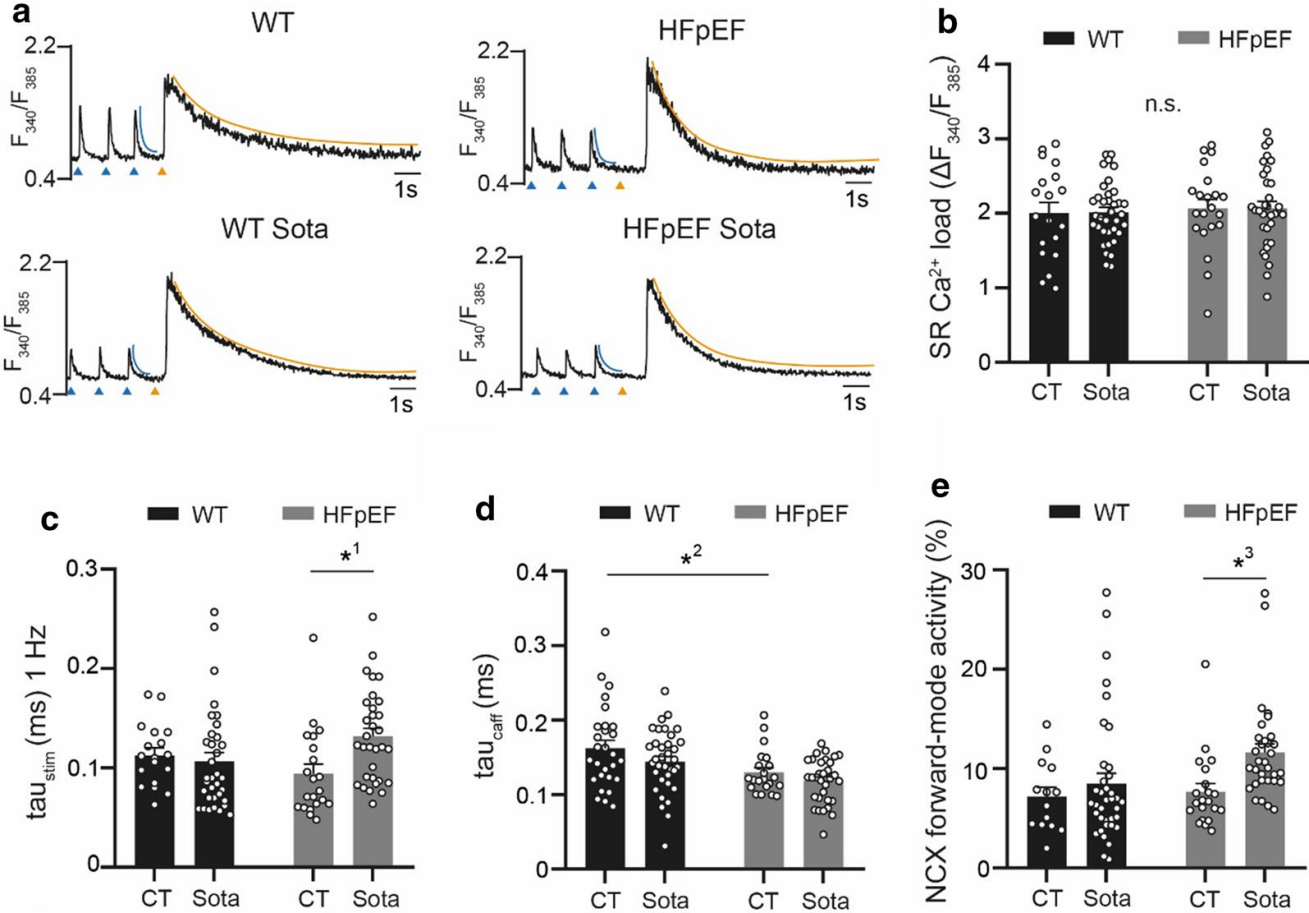
**a** Representative examples of electrically (1 Hz; left) and caffeine-induced (20 mM; right) CaT. Tau of decay was determined during electric stimulation (left; blue curvature) and after caffeine (right; orange curvature). Blue arrows indicate electric stimulation triggers, orange arrows indicate caffeine application. Related data of **b** SR Ca^2+^ load, **c** tau of electrically stimulated CaT (**d**), tau of caffeine-induced CaT and **e** NCX forward-mode activity (calculated from **c** and **d**, see “Methods”). Statistical analysis: two-way ANOVA followed by post-hoc Bonferroni. *p*-values: ^1^0.008, ^2^0.01, ^3^0.01. n = cells derived from 6 animals per group

20 mM caffeine was added to Tyrode solution to induce sarcoplasmic reticulum (SR) Ca^2+^ release and obtain a measure of SR Ca^2+^ content (Fig. 2f, g). For baseline recordings of Ca^2+^ transient (CaT), sarcomere shortening and arrhythmic events (Figs. 1c–f, 2a–e, 3a–f), Tyrode solution containing 3 mM Ca^2+^ was used. For glucose starvation (Figs. 2h–j, 5a–d), glucose of Tyrode solution was replaced with 30 mM mannitol. For sub-sequent glucose reintroduction, mannitol was replaced with 30 mM glucose (“high glucose condition”).

For cell membrane permeabilization and subsequent measurements of mitochondrial Ca^2+^ uptake (see “Mitochondrial structure and Ca^2+^ uptake”) wash and internal solutions were used*.* Wash solution contained (in mM): 100 potassium acetate, 15 KCl, 0.35 EGTA, 0.75 MgCl_2_, 10 HEPES; pH adjusted to 7.2 with KOH. Internal solution contained (in mM): 125 KCl, 10 NaCl, 20 HEPES, 5 pyruvate, 2 maleic acid, 2 glutamic acid, 0.5 KH_2_PO_4_, 0.5 MgCl_2_, 5 EGTA, 0.002 free Ca^2+^ (MaxChelator), 15 BDM, pH adjusted to 7.2 with KOH. For permeabilization, 0.005% saponin was added to the internal solution.

Fluorescent dyes were used at the following concentration (in mM): 0.002 Fura, 1 Rhod-2, 1 Mitotracker, 0.01 H2-DCF, 50^–6^ TMRE.

For Western blot analysis, LA tissue was homogenized in lysis buffer. Lysis buffer contained (in mM): 137 NaCl, 20 NaF, 1 sodium pyrophosphate, 50 β-glycerophosphate, 10 EDTA, 1 EGTA, 1 PMSF, 10% glycerol, 1% NP 40, 4 µg/ml aprotinin 4 µg/ml pepstatin A, 4 µg/ml leupeptin.

### Ca^2+^ measurements and sarcomere shortening

**Fig. 4:**
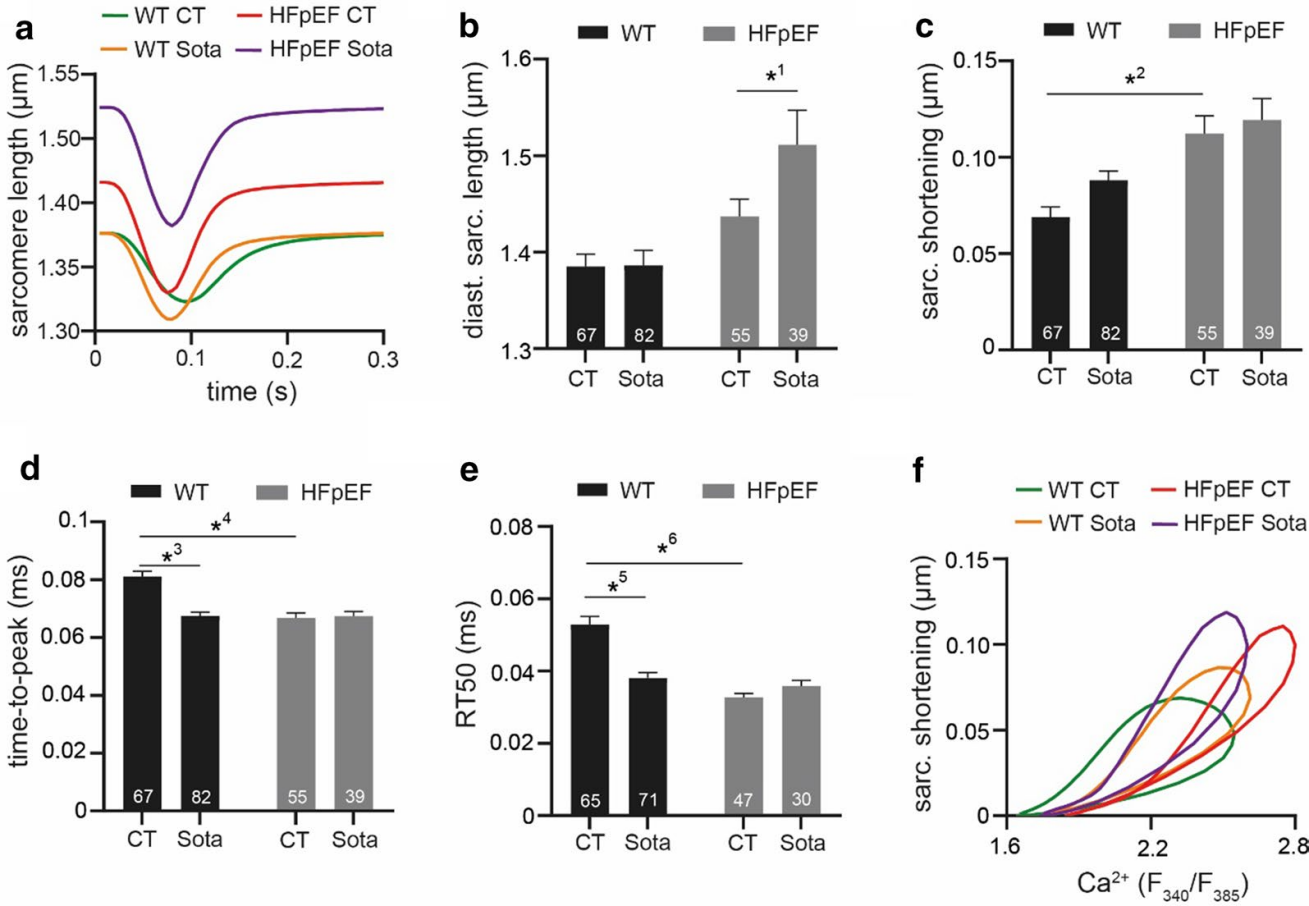
**a** Representative examples of sarcomere shortening during 3 Hz electric stimulation and 5 mM extracellular [Ca^2+^]. Related data of **b** diastolic sarcomere length, **c** sarcomere shortening, **d** time-to-peak and **e** RT50 of decay. **f** Relationship of averaged CaT (Fig. 2a) and averaged sarcomere shortening (Fig. 3a). Statistical analysis: two-way ANOVA followed by post-hoc Bonferroni. *p*-values: ^1^0.03, ^2^< 0.001, ^3^< 0.001, ^4^< 0.001, ^5^< 0.001, ^6^< 0.001. n = cells derived from 6 animals per group

Ratiometric Ca^2+^ measurements (excitation: 340 nm and 385 nm, emission: 510 ± 10 nm; [Ca^2+^] expressed as the ratio *R* = F_340_/F_380_) were performed either with a CytoCypher MultiCell System (CytoCypher BV, Netherlands; Figs. 1c–f, 2d–g, 4a–f) or an Axiovert 200 microscope (Zeiss, Oberkochen, Germany) fluorescence imaging assembly (Figs. 2a–c, 2h–j, 3a–e, 6e–i). LA cardiomyocytes were loaded with Fura-2 for 30 min at room temperature, washed twice with Tyrode solution and transferred to the microscope. Experiments were conducted at 37 °C and CaT were recorded at steady-state (following electric stimulation).

CaT and sarcomere shortening of LA and LV cardiomyocytes were recorded for 10 s at 3 Hz stimulation (Figs. 2d–g, 4a–f). Electric stimulation was turned off and spontaneous SR Ca^2+^ release events [24] (SCaEs) immediately recorded for a duration of 10 s (Fig. 1c–f). In a sub-set of LA cells, CaT were recorded for 10 s at 1 Hz stimulation. Electric stimulation was turned off, the cells immediately exposed to 20 mM caffeine and the sub-sequent caffeine-induced CaT recorded for 10 s (Figs. 2a–c, 3a–e). Sodium–calcium exchanger (NCX) activity was calculated as previously described [25].

**Fig. 5:**
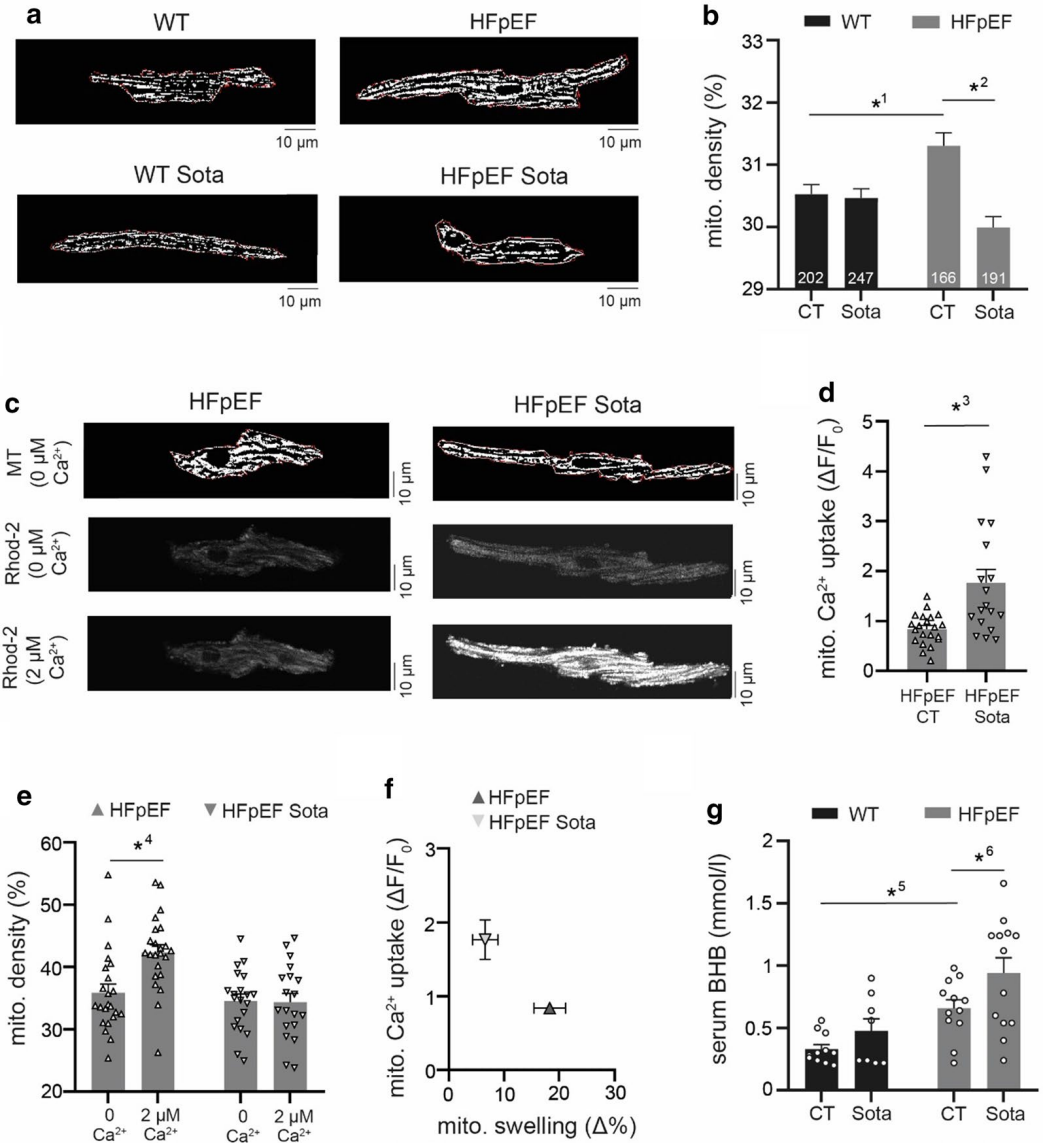
**a** Representative examples of mitochondrial structure in LA cardiomyocytes (dye: MitoTracker after thresholding) and **b** related data of mitochondrial density. **c** Representative example of mitochondrial structure of LA cardiomyocytes after permeabilization of the sarcolemma (above), mitochondrial Ca^2+^ during perfusion with internal solution containing either 0 µM (center) or 2 µM Ca^2+^ (below). **d** Related data of mitochondrial Ca^2+^ uptake, **e** mitochondrial density and **f** correlation of mitochondrial Ca^2+^ uptake and mitochondrial swelling. **g** Serum concentration of β-hydroxybutyrate (BHB). Statistical analysis: Two-way ANOVA followed by post-hoc Bonferroni (**b**, **e**, **g**) or unpaired, two-tailed Student’s t-test (**d**). *p*-values: ^1^0.004, ^2^< 0.001, ^3^< 0.001, ^4^< 0.001, ^5^0.026, ^6^0.049. **a**–**f** n = cells derived from 6 animals per group. **g** n = animals

For measurement under different metabolic conditions, LA cardiomyocyte CaT were recorded for 10 s at 1 Hz (Fig. 5e–i). Cells were treated with 2-deoxyglucose to inhibit glycolysis for a duration of 3 min, while maintaining steady stimulation at 1 Hz and CaT were recorded for another 10 s. A sub-set of cells was starved of glucose for 1 h at 37 °C. CaT were recorded for 10 s at 1 Hz stimulation (Fig. 2h–j). Cells were exposed to glucose and constant electric pacing at 1 Hz was maintained. After 1 min, CaT transients were recorded for 10 s at 1 Hz stimulation.

### Mitochondrial structure and Ca^2+^ uptake

**Fig. 6:**
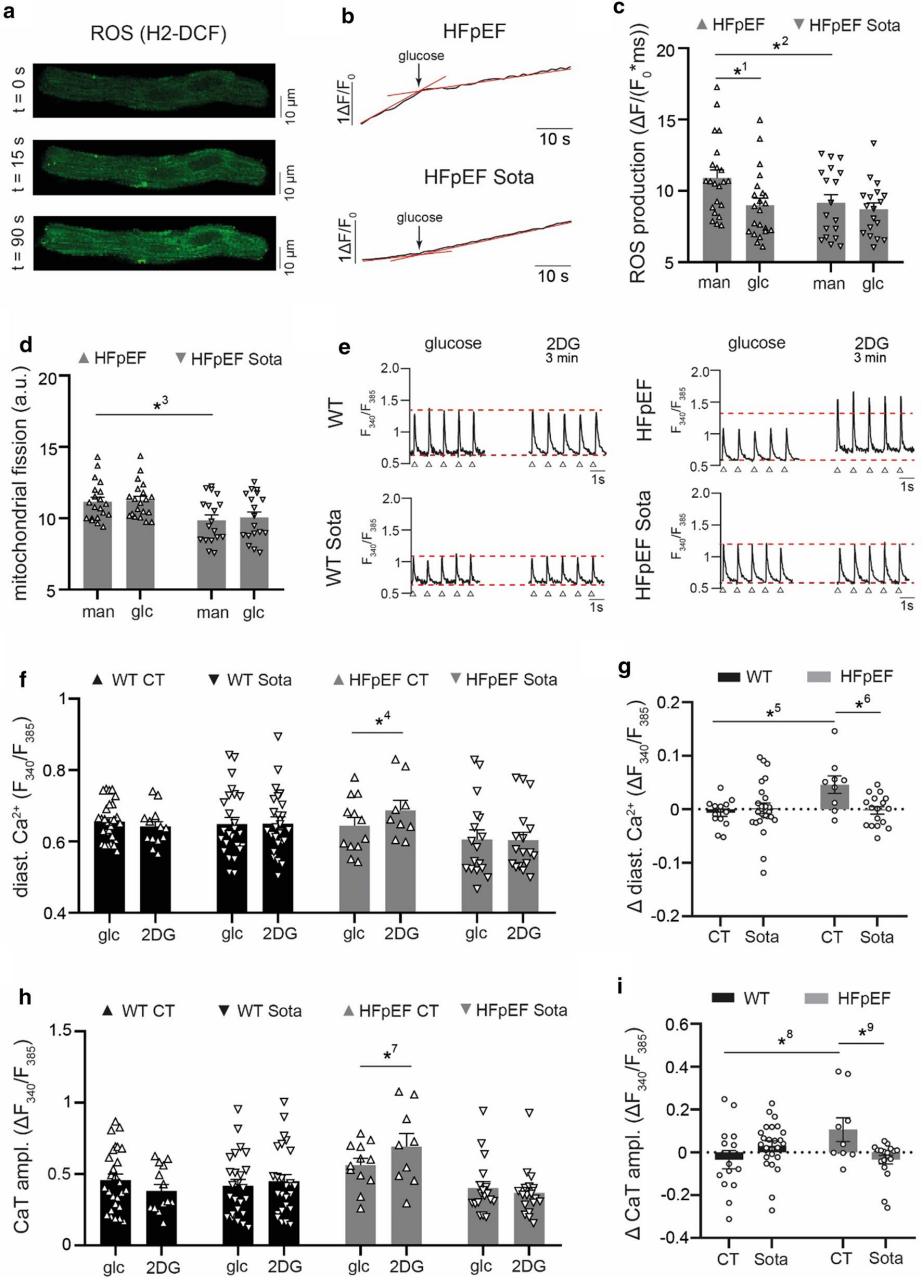
**a** Example image sequence of ROS measurements in LA cardiomyocytes (shown: HFpEF). **b** Representative example and **c** related data of ROS production after 1 h incubation in glucose-deprived buffer (man) and after 30 s of glucose reintroduction (glc). **d** Mitochondrial fission of LA cardiomyocytes after 1 h incubation in glucose-deprived buffer (man) and after 60 s of glucose reintroduction (glc). **e** Representative examples of CaT at 1 Hz electric stimulation before (left) and 3 min after glycolytic inhibition with 2-deoxyglucose (2DG; right). Red, dashed lines indicate CaT peak (upper) and diastolic Ca^2+^ (lower) at baseline, arrows indicate stimulation triggers. Related data of **f** diastolic Ca^2+^ and (H) CaT amplitude before (glc) and after glycolytic inhibition (2DG); **g** corresponding change of diastolic Ca^2+^ and (I) CaT amplitude. Statistical analysis: Two-way ANOVA followed by post-hoc Bonferroni. *p*-values: ^1^< 0.001, ^2^0.046, ^3^0.015, ^4^0.005, ^5^0.007, ^6^0.01, ^7^0.04, ^8^0.02, ^9^0.016. n = cells derived from 6 animals per group

Mitochondrial structure was visualized by local thresholding of two-dimensional images acquired with MitoTracker Red (Figs. 5a–b, 6d) and MitoTracker Green (Fig. 4c–f) at an LSM 800 laser scanning microscope (Zeiss, Oberkochen, Germany). The fraction of mitochondria in relation to cell surface was taken as a measure of mitochondrial density. The averaged perimeter to area ratio of mitochondrial structures per cell was calculated as an indicator of mitochondrial fission using a 2-step Otsu thresholding algorithm [26].

Mitochondrial Ca^2+^ uptake was determined as previously described in detail [27]. LA cardiomyocytes were loaded with Rhod-2 AM and MitoTracker green, transferred to an LSM 800 laser scanning microscope and washed twice with sodium and calcium-free wash solution. The cells were then permeabilized with internal solution containing 0.005% saponin for a duration of 30–60 s and consecutively washed twice with nominal Ca^2+^ free internal solution containing 5 mM EGTA. Two-dimensional images of Rhod-2 (excitation: 559 nm, emission: 575–675 nm) and MitoTracker green fluorescence (excitation: 488 nm, emission: 505–525 nm) were obtained. The perfusion was switched to internal solution containing 2 µM Ca^2+^. After 1 min, a second set of Rhod-2/MitoTracker images was obtained. Following another 1-min interval, a third set of images was obtained to confirm that mitochondrial Ca^2+^ uptake had indeed been completed in the second set. A binary mask of mitochondrial structures was derived from MitoTracker green images and positive pixels defined as the region-of-interest for sub-sequent determination of Rhod-2 signal intensity (F). Signal intensity during perfusion with 0 µM Ca^2+^ was defined as F_0_ and changes of [Ca^2+^] after exposure to 2 µM Ca^2+^ expressed as ΔF = F—F_0_. The change of mitochondrial density (Δ%) was quantified as a measure of mitochondrial swelling.

### Mitochondrial depolarization

LA cardiomyocytes were loaded with TMRE and MitoTracker green, transferred to an LSM 800 laser scanning microscope and kept in Tyrode’s solution containing 2 mM Ca^2+^ and 10 nM TMRE. Two-dimensional images of TMRE (excitation: 561 nm, emission: 565–585 nm) and MitoTracker green fluorescence (excitation: 488 nm, emission: 505–525 nm) were acquired for a duration of 6 min (interval: 2 s, resolution: 512 × 512 px, pixel size: 1.25 µm, pixel time: 1.03 µs, laser intensity: 4%). A binary mask of both channels was derived using a Bernsen thresholding algorithm (ImageJ). Positive pixels of the MitoTracker green image were defined as mitochondria and a positive overlay of the TMRE image assumed to indicate a polarized state. The standard deviation of polarized mitochondria over time was taken as a measure of spatiotemporal oscillation.

### ROS production

LA cardiomyocytes were starved of glucose for 1 h at 37 °C, loaded with H2-DCF and transferred to an LSM 800 laser scanning microscope. Two-dimensional images (excitation: 488 nm, emission: 505–252 nm) were acquired for a duration of 30 s (interval: 2 s, resolution: 256 × 256 px, pixel size: 0.624 µm, pixel time: 8.24 µs, laser intensity: 0.6%). Cells were exposed to 30 mM glucose and another set of images acquired for a duration of 90 s. Image sequences acquired between 0–30 s (glucose starved) and 90–120 s (glucose saturated) were individually assessed. H2-DCF signal intensity (F) of the initial image was defined as F_0_, reactive oxygen species (ROS) accumulation calculated as ΔF = F − F_0_ per image, averaged per image sequence and reported as the respective rate ΔF/(F_0_ * t).

### Western Blots

LA tissue homogenate was run on a 4–12% Bis–Tris gel and transferred to a 0.45 µm nitrocellulose membrane for 120 min. The total protein on the membrane was stained with Ponceau S. Non-specific binding was blocked with 5% dried milk in Tris-buffered saline (pH 7.4) containing 0.1% Tween-20. Membranes were probed with anti-SGLT-1 (biomol, Germany), anti-SGLT-2 (abcam, UK & Santa Cruz, CA, US). Anti-mouse IgG linked with IRDye 680RD or anti-rabbit IgG linked with 800CW (LI-COR) were used as a secondary antibody. The signal was recorded with an Odyssey CLx System. Band intensities and total protein were determined by Image Studio software (LI-COR).

### Data analysis and statistics

Results are shown as mean ± standard error. Individual data points are shown where spatially feasible. Statistical tests and *p*-values are supplied for each graph in the figure legend. A *p*-value of < 0.05 was considered to be of statistical significance.

## Results

### *LA/LV interaction and Sota mitigating left atrial enlargement and arrhythmic Ca*^*2*+^*release in HFpEF*

*In-vivo* LA volume obtained via echocardiography showed severely enlarged atria in the HFpEF group (Fig. 1a, b). LA enlargement correlated with LV function (Additional file 1: Figure S1). In-vitro, LV and LA cardiomyocytes correlated regarding diastolic [Ca^2+^] (R^2^ = 0.98) and regarding (the closely related) diastolic sarcomere length (R^2^ = 0.63), indicative of LV/LA interaction in this HFpEF atrial cardiomyopathy model (Additional file 1: Figure S2). Spontaneous Ca^2+^ release events (SCaEs) of LA cardiomyocytes were more frequent and their Ca^2+^ release amplitude increased in HFpEF (Fig. 1c–e). Sota mitigated LA enlargement in HFpEF. Even though the event frequency remained unaltered, the amplitude of SCaEs in HFpEF was significantly reduced following Sota treatment. Overall, LA volume in-vivo correlated with the occurrence of SCaEs *in-vitro*, indicating mechanical stretch of cardiomyocytes (as determined by volumetric load *in-vivo*) to be a potential modulator of arrhythmic SR Ca^2+^ release in this model (Fig. 1f).

### *Sota lowers diastolic Ca*^*2*+^*in LA cardiomyocytes in HFpEF*

First, we examined CaT of LA cardiomyocytes at 1 Hz stimulation and 2 mM extracellular [Ca^2+^] (Fig. 2a). No differences could be observed in diastolic Ca^2+^ and CaT amplitude in HFpEF *vs.* WT (Fig. 2b, c). We then challenged the cells with increased stimulation frequencies (3 Hz) and extracellular [Ca^2+^] (5 mM; Fig. 2d). Again, we could not observe a difference in diastolic Ca^2+^, however, CaT amplitudes in HFpEF were increased (Fig. 2e, f). Time-to-peak remained unchanged (Fig. 2g). In order to assess the effect of glucose influx on cytosolic [Ca^2+^], LA cardiomyocytes were starved of glucose for 1 h in Tyrode’s solution containing 30 mM mannitol and consecutively challenged with 30 mM glucose (Fig. 2h). Both HFpEF and WT showed an increase in diastolic [Ca^2+^] at a similar extent (Fig. 2i, j). Chronic treatment with Sota did not alter diastolic [Ca^2+^] and CaT amplitude at baseline (1 Hz, 2 mM Ca^2+^). When challenged with 3 Hz and 5 mM extracellular [Ca^2+^] however, Sota lead to a significant reduction of diastolic [Ca^2+^] in HFpEF, while leaving CaT amplitudes unchanged. Interestingly, Sota also prevented glucose-mediated influx of diastolic Ca^2+^ in HFpEF.

### Sota increases NCX forward-mode activity in HFpEF

CaT of LA cardiomyocytes were recorded during electric stimulation and after the application of caffeine in order to assess SR Ca^2+^ load, as well as the relative contribution of NCX activity towards cytosolic Ca^2+^ removal (Fig. 3a). In HFpEF, SR Ca^2+^ load and tau of decay during paced CaT remained unchanged (Fig. 3b, c). Tau of decay of caffeine-induced CaT however was significantly shorter in HFpEF (Fig. 3d). The contribution of NCX forward-mode activity to cytosolic [Ca^2+^] removal in paced CaT was unaltered in HFpEF (Fig. 3e). Treatment with Sota had no effect on SR Ca^2+^ load in HFpEF. In HFpEF, tau of decay was significantly prolonged in with Sota, yet tau of caffeine-induced CaT was unchanged. Interestingly, this resulted in a ~ 50% increased contribution of NCX forward-mode activity on cytosolic Ca^2+^ removal (7.6 ± 0.7 vs. 11.6 ± 0.7%, n = 14 and 21 cells).

### Sota lengthens sarcomeres during diastole in HFpEF

Next, we investigated the effect of Sota on cardiomyocyte mechanics (Fig. 4a). Diastolic sarcomere length remained unaltered in HFpEF vs. WT. In support of the notion of a rather compensatory atrial phenotype [21], HFpEF cardiomyocytes showed an increased sarcomere shortening, shorter time-to-peak and relaxation time (Fig. 4c–e) vs. WT. Sota led to a significant increase of diastolic sarcomere length in HFpEF. In WT, Sota shortened time-to-peak and relaxation time, while this effect could not be observed in HFpEF. Overall, Sota reduced Ca^2+^ sensitivity in HFpEF (Fig. 4f).

### *Sota prevents mitochondrial swelling and increases mitochondrial Ca*^*2*+^*uptake in HFpEF*

To further elucidate how Sota mitigated atrial *in-vivo* remodeling and decreased the propensity for pro-arrhythmic Ca^2+^ release, we measured mitochondrial structure (Fig. 5a, b) and Ca^2+^ uptake (Fig. 5c, d). An increased density of mitochondria in LA cardiomyocytes could be observed in HFpEF, which was prevented by Sota. Treatment with Sota led to a two-fold increase of mitochondrial Ca^2+^ uptake in HFpEF (0.84 ± 0.07 vs. 1.76 ± 0.27 ΔF/F_0_, n = 21 and 18 cells from 6 animals/group) in permeabilized cells after exposure from 0 µM to 2 µM Ca^2+^. Additionally, a notable swelling of mitochondria was visible in HFpEF cells (35.9 ± 1.8 to 42.4 ± 1.6%, n = 21 cells and 18 cells from 6 animals), while this effect did not occur with Sota (Fig. 5e, f). Analysis of circulating ketone bodies revealed a shift in the availability of mitochondrial fuel: HFpEF showed an increased concentration of β-hydroxybutyrate compared to the control group, which was even further enhanced by Sota treatment (Fig. 5g). Differences in the incidence and spatial distribution of mitochondrial depolarizations could not be detected (Additional file 1: Figure S3).

### Sota improves metabolic dysfunction during glucose depletion in HFpEF

As impaired myocardial glucose metabolism and increased oxidative stress are hallmark features of heart failure and acute decompensation [28], we used glucose depletion to further challenge HFpEF cardiomyocytes. Sota significantly reduced ROS production (Fig. 6a–c) and mitochondrial fission of LA cardiomyocytes after 1 h glucose starvation in HFpEF (Fig. 6d). As anticipated, ROS production in HFpEF decreased after reintroduction of glucose, while this effect could not be observed with Sota. In line with this, Sota also prevented an increased influx of diastolic Ca^2+^ and an increased CaT amplitude gain upon glycolytic inhibition with 2-deoxyglucose in HFpEF (Fig. 6e–i). Under baseline conditions, antioxidative treatment with acetylcysteine decreased the occurrence of SCaEs in LA cardiomyocytes in both HFpEF groups (Additional file 1: Figure S4).

## Discussion

Chronic treatment with the dual SGLT-1&2 inhibitor sotagliflozin was effective in mitigating LA cardiomyopathy in a rat model of metabolic syndrome-related HFpEF. In HFpEF, Sota decreased the magnitude of arrhythmic Ca^2+^ release events of LA cardiomyocytes *in-vitro.* Sota reduced cytosolic [Ca^2+^] at baseline, as well as in response to glucose influx and depletion. Lower cytosolic [Ca^2+^] was accompanied by an increased Ca^2+^ buffer capacity of the mitochondrial compartment, decreased mitochondrial swelling at baseline and lower ROS production during glucose depletion.

In human right atria, previous work by Voigt et al*.* has highlighted the role of pro-arrhythmic SCaEs of cardiomyocytes in persistent AF [24]. The authors describe an increased SCaE incidence and Ca^2+^ release amplitude, accompanied by alterations of intrinsic Ca^2+^ cycling, i.e. enhanced SERCA function, increased CaT amplitude, larger RyR-mediated Ca^2+^ leak and unaltered NCX activity. The present model is not known to be a dedicated AF model and we did not find overt AF during our final experiments. However, atrial remodeling and atrial cardiomyopathy are entities preceding the presence of AF [3]. In support of this notion, our current study and previous work showed an overall similar cellular phenotype regarding Ca^2+^ handling in HFpEF-related LA remodeling [21]. This indicates a common denominator of pro-arrhythmogenic atrial remodeling, potentially associated with a progression towards AF. Chronic dual SGLT-1&2 inhibition led to a reduction of SCaE amplitudes in HFpEF, yet the incidence of events remained unaffected (Fig. 1e, f). This observation can be explained by an increased NCX forward-mode activity: Enhanced Ca^2+^ extrusion mitigates cytosolic Ca^2+^ overload (i.e. ryanodine receptor-mediated leak) and unburdens intrinsic Ca^2+^ buffer systems (i.e. mitochondria). This potentially alleviates pro-arrhythmic organ wide events as it also impacts Ca^2+^ wave propagation and limits spontaneous cytosolic Ca^2+^ induced Ca^2+^ release[29]. However, increased forward-mode activity also leads to a positive net charge shift (1 Ca^2+^ outwards, 3 Na^+^ inwards), which has been associated with an increased frequency of triggered, arrhythmic Ca^2+^ release events in patients with AF [30]. Recent data linked increased reverse mode NCX activity in ventricular cardiomyocytes to cardiac remodeling and diastolic dysfunction in a rat model of HFpEF (following partial nephrectomy) [31]. In contrast, in atrial cardiomyocytes, increased forward mode NCX was a potential contributor to the amelioration of structural remodeling (e.g. LA enlargement) observed in this study. Interestingly, increased forward mode NCX activity after chronic treatment with Sota only occurs in HFpEF, but not WT. Even though intracellular [Na^+^] was not determined in the current study, a probable driver might be a reduction of (initially elevated) cytosolic [Na^+^]. Different mechanisms of [Na^+^] lowering seem plausible: SGLT-2 inhibitors have been demonstrated to inhibit the Na^+^/H^+^ exchanger in murine cardiomyocytes [32]. Indeed, our results confirm not only the debated presence of SGLT-2 in the LA [11] but also support the notion of altered cytosolic [Na^+^] and [Ca^2+^] by its inhibition. Work by Lambert et al*.* indicates a contribution of the SGLT-1 transporter to cytosolic [Na^+^] in failing hearts, in particular in the presence of metabolic dysfunction (T2DM, obesity), making it another plausible site-of-action [14].

Mitochondria sequester large amounts of Ca^2+^, which is a crucial regulator of energy production, mitochondrial morphology and apoptosis. In the ZSF model of HFpEF, an elevated mitochondrial [Ca^2+^] of LV cardiomyocytes at rest has been associated with increased cytosolic [Ca^2+^], mitochondrial swelling and reduced mitochondrial respiration [20]. In our study, SGLT-1&2 inhibition normalized abnormal mitochondrial swelling of LA cardiomyocytes in HFpEF and enhanced mitochondrial Ca^2+^ buffer capacity (Fig. 5). This effect might be explained by a reduction of mitochondrial [Ca^2+^] at rest through reduced cytosolic [Na^+^] or [Ca^2+^] [33]. Mitochondrial Ca^2+^ uptake has been shown to contribute to the buffering of cytosolic Ca^2+^ peaks in cardiomyocytes [25, 34] and pharmacologic enhancement of mitochondrial Ca^2+^ uptake was associated with decreased SCaEs in catecholaminergic ventricular tachycardia models [35]. An increased mitochondrial Ca^2+^ buffer capacity might therefore contribute to decreased SCaEs amplitudes. Mitochondrial swelling has been described as a consequence of [Ca^2+^] overload, consecutively leading to an opening of the mitochondrial permeability transition pore, mitochondrial depolarization, ROS generation and ultimately apoptosis [36]. We further explored ROS-dependent SCaEs and spatial aspects of mitochondrial depolarizations, both established mediators of cellular arrhythmias [37]. However, we were unable to detect an effect with dual SGLT-1&2 inhibition (Additional file 1: Figure S3, S4), indicating that altered NCX activity and Ca^2+^ buffer related mechanisms are of greater relevance for the observed Sota-related reverse atrial remodeling.

A reduced cardiac energy reserve and metabolic disorders are hallmark features of severe HF. In addition, almost 50% of HFpEF patients suffer from T2DM and are at particular high risk for HF hospitalization [38]. SGLT inhibition and in particular Sota have been shown to provide beneficial effects on blood pressure and body weight in the setting of diabetes potentially through reduced glycogen accumulation and ROS production [39, 40]. While dual SGLT inhibition has also been associated with an exacerbation of cardiac dysfunction following myocardial infarction [41] in line with enhanced SGLT-1 mediated oxidative stress [42], others reported a protective role of SGLT-1 during the acute phase of ischemia/reperfusion injury [43]. Cardiac hypertrophy, a common predecessor of HFpEF, has frequently been linked to an increased glycolytic and decreased mitochondrial capacity [44, 45]. Recent animal studies suggest an additional uncoupling of glycolysis from mitochondrial glucose oxidation in HFpEF [46]. Work by Yoshii et al. has shown the significant role of SGLT-1 in the myocardial glucose uptake of the diabetic heart with respect to other glucose transporters (GLUT4 and GLUT1) [43] and altered (mitochondrial) Ca^2+^ homoeostasis is an established regulator of cellular energetics [47]. Moreover, Empagliflozin has been shown to mitigate diabetes related atrial fibrillation via improved mitochondrial function [48]. We therefore investigated whether Sota would normalize glucose-mediated metabolic abnormalities related to cellular arrhythmogenesis of LA cardiomyocytes in HFpEF (e.g. Ca^2+^ cycling, ROS production). Indeed, Sota prevented cytosolic Ca^2+^ accumulation upon glucose influx and glycolytic inhibition in HFpEF. Dual SGLT-1&2 inhibition lowered ROS production during glucose starvation. Interestingly, ROS production normalized upon reintroduction of glucose only in HFpEF, indicating an increased glucose-dependency to meet cellular energetic demand while maintaining an adequate degree of pro-arrhythmogenic ROS production.

## Conclusion

The dual SGLT-1&2 inhibitor sotagliflozin ameliorated LA remodeling in HFpEF and exerted an anti-arrhythmic effect on LA cardiomyocytes. The safety and efficacy of dual SGLT-1&2 inhibition for the treatment and/or prevention of AF in HFpEF should be further evaluated in clinical trials.

## Supplementary Information

**Additional file 1: Figure S1.** (A) Longitudinal and (B) transversal LV fractional shortening as observed in echocardiography (parasternal long axis view). (C) Correlation of longitudinal and (D) transversal LV fractional shortening with LA volume. (E) Exemplary electrocardiogram to monitor atrial fibrillation (excerpt of 30 min continuous recording). (F) Total occurrence of atrial fibrillation during screened time intervals. (G) Serum concentration of creatinine and (H) urea. Statistical analysis: Two-way ANOVA followed by post-hoc Bonferroni (G, H). n = animals. ^1^0.016, ^2^ < 0.001, ^3^ < 0.001, ^4^0.014, ^5^ < 0.001. **Figure S2.** (A) Correlation of diastolic Ca^2+^, (B) CaT amplitudes, (C) CaT time-to-peak (TTP), (D) CaT tau of decay, (E) diastolic sarcomere length, (F) sarcomere shortening, (G) sarcomere time-to-peak (TTP) and sarcomere RT50 (H) of LV and LA cardiomyocytes. *p*-values (deviation from zero): ^1^ < 0.0001, ^2^0.005, ^3^0.01. n = average per animal. **Figure S3.** (A) Western Blot of LA SGLT-1 expression (above) and respective total protein visualized by Ponceau S staining (below). (B) Corresponding statistical analysis of SGLT-1 signal intensity after normalization to total protein. (C) Western Blot of LA SGLT-2 expression (above). and respective total protein (below), kidney (K) shown as positive control. (D) Corresponding SGLT-2 signal intensity after normalization to total protein. Statistical analysis: Two-way ANOVA followed by Fisher’s LSD test. n = animals. ^1^0.003, ^2^0.048, ^3^0.03. **Figure S4.** (A) Example of polarized mitochondria (TMRE / MitoTracker binary overlay after local thresholding) at baseline (left) and after spontaneous depolarization (right) and corresponding signal trace (below). (B) Standard deviation (SD) of signal trace over the course of 6 min. (C) Maximum depolarization per cell. Statistical analysis: Two-way ANOVA followed by post-hoc Bonferroni. **Figure S5.** (A) Example of SCaEs during a non-stimulated interval, following 3 Hz stimulation for 10 s at baseline (above) and after 10 min incubation with acetylcysteine (ACC; below). (B) Related occurrence of SCaEs (all HFpEF). Statistical analysis: Two-way ANOVA followed by post-hoc Bonferroni. *p*-values: ^1^0.069, ^2^0.018.

## Data Availability

The datasets used and/or analyzed during the current study are available from the corresponding author on reasonable request.

## Abbreviations

AF: Atrial fibrillation
CaT: Ca^2^^+^ transient
EF: Ejection fraction
HF: Heart failure
HFpEF: Heart failure with preserved ejection fraction
HFrEF: Heart failure with reduced ejection fraction
LA: Left atrium/left atrial
NCX: Sodium–glucose exchanger
ROS: Reactive oxygen species
SCaE: Sarcoplasmic reticulum Ca^2^^+^ release events
SGLT: Sodium–glucose linked transporter
Sota: Sotagliflozin
SR: Sarcoplasmic reticulum
T2DM: Diabetes mellitus type 2
WT: Wild type

## References

[1] Hohendanner F, Messroghli D, Bode D, Blaschke F, Parwani A, Boldt LH, Heinzel FR (2018). Atrial remodelling in heart failure: recent developments and relevance for heart failure with preserved ejection fraction. ESC Heart Fail.

[2] Hohendanner F, Heinzel FR, Blaschke F, Pieske BM, Haverkamp W, Boldt HL, Parwani AS (2018). Pathophysiological and therapeutic implications in patients with atrial fibrillation and heart failure. Heart Fail Rev.

[3] Goette A, Kalman JM, Aguinaga L, Akar J, Cabrera JA, Chen SA, Chugh SS, Corradi D, D'Avila A, Dobrev D (2016). EHRA/HRS/APHRS/SOLAECE expert consensus on Atrial cardiomyopathies: Definition, characterisation, and clinical implication. J Arrhythm.

[4] Marrouche NF, Brachmann J, Andresen D, Siebels J, Boersma L, Jordaens L, Merkely B, Pokushalov E, Sanders P, Proff J (2018). Catheter ablation for atrial fibrillation with heart failure. N Engl J Med.

[5] Zelniker TA, Bonaca MP, Furtado RHM, Mosenzon O, Kuder JF, Murphy SA, Bhatt DL, Leiter LA, McGuire DK, Wilding JPH (2020). Effect of dapagliflozin on atrial fibrillation in patients with type 2 diabetes mellitus: insights from the DECLARE-TIMI 58 trial. Circulation.

[6] McMurray JJV, Solomon SD, Inzucchi SE, Kober L, Kosiborod MN, Martinez FA, Ponikowski P, Sabatine MS, Anand IS, Belohlavek J (2019). Dapagliflozin in patients with heart failure and reduced ejection fraction. N Engl J Med.

[7] Abdul-Ghani MA, DeFronzo RA, Norton L (2013). Novel hypothesis to explain why SGLT2 inhibitors inhibit only 30–50% of filtered glucose load in humans. Diabetes.

[8] de Boer RA, Nunez J, Kozlovski P, Wang Y, Proot P, Keefe D (2020). Effects of the dual sodium-glucose linked transporter inhibitor, licogliflozin vs placebo or empagliflozin in patients with type 2 diabetes and heart failure. Br J Clin Pharmacol.

[9] Bonora BM, Avogaro A, Fadini GP (2020). Extraglycemic effects of SGLT2 inhibitors: a review of the evidence. Diabetes Metab Syndr Obes.

[10] Zambrowicz B, Freiman J, Brown PM, Frazier KS, Turnage A, Bronner J, Ruff D, Shadoan M, Banks P, Mseeh F (2012). LX4211, a dual SGLT1/SGLT2 inhibitor, improved glycemic control in patients with type 2 diabetes in a randomized, placebo-controlled trial. Clin Pharmacol Ther.

[11] Sabatino J, De Rosa S, Tamme L, Iaconetti C, Sorrentino S, Polimeni A, Mignogna C, Amorosi A, Spaccarotella C, Yasuda M (2020). Empagliflozin prevents doxorubicin-induced myocardial dysfunction. Cardiovasc Diabetol.

[12] Powell DR, Zambrowicz B, Morrow L, Beysen C, Hompesch M, Turner S, Hellerstein M, Banks P, Strumph P, Lapuerta P (2020). Sotagliflozin decreases postprandial glucose and insulin concentrations by delaying intestinal glucose absorption. J Clin Endocrinol Metab.

[13] Kashiwagi Y, Nagoshi T, Yoshino T, Tanaka TD, Ito K, Harada T, Takahashi H, Ikegami M, Anzawa R, Yoshimura M (2015). Expression of SGLT1 in human hearts and impairment of cardiac glucose uptake by phlorizin during ischemia-reperfusion injury in mice. PLoS ONE.

[14] Lambert R, Srodulski S, Peng X, Margulies KB, Despa F, Despa S (2015). Intracellular Na+ concentration ([Na+]i) Is Elevated In Diabetic Hearts Due To Enhanced Na+-glucose cotransport. J Am Heart Assoc.

[15] Seidelmann SB, Feofanova E, Yu B, Franceschini N, Claggett B, Kuokkanen M, Puolijoki H, Ebeling T, Perola M, Salomaa V (2018). Genetic variants in SGLT1, glucose tolerance, and cardiometabolic risk. J Am Coll Cardiol.

[16] Banerjee SK, McGaffin KR, Pastor-Soler NM, Ahmad F (2009). SGLT1 is a novel cardiac glucose transporter that is perturbed in disease states. Cardiovasc Res.

[17] Bilan VP, Salah EM, Bastacky S, Jones HB, Mayers RM, Zinker B, Poucher SM, Tofovic SP (2011). Diabetic nephropathy and long-term treatment effects of rosiglitazone and enalapril in obese ZSF1 rats. J Endocrinol.

[18] Hamdani N, Franssen C, Lourenco A, Falcao-Pires I, Fontoura D, Leite S, Plettig L, Lopez B, Ottenheijm CA, Becher PM (2013). Myocardial titin hypophosphorylation importantly contributes to heart failure with preserved ejection fraction in a rat metabolic risk model. Circ Heart Fail.

[19] Bowen TS, Brauer D, Rolim NPL, Baekkerud FH, Kricke A, Ormbostad Berre AM, Fischer T, Linke A, da Silva GJ, Wisloff U (2017). Exercise training reveals inflexibility of the diaphragm in an animal model of patients with obesity-driven heart failure with a preserved ejection fraction. J Am Heart Assoc.

[20] Miranda-Silva D, Wust RCI, Conceicao G, Goncalves-Rodrigues P, Goncalves N, Goncalves A, Kuster DWD, Leite-Moreira AF, van der Velden J, de Sousa Beleza JM (2020). Disturbed cardiac mitochondrial and cytosolic calcium handling in a metabolic risk-related rat model of heart failure with preserved ejection fraction. Acta Physiol (Oxf).

[21] Hohendanner F, Bode D, Primessnig U, Guthof T, Doerr R, Jeuthe S, Reimers S, Zhang K, Bach D, Wakula P (2018). Cellular mechanisms of metabolic syndrome-related atrial decompensation in a rat model of HFpEF. J Mol Cell Cardiol.

[22] Powell DR, DaCosta CM, Smith M, Doree D, Harris A, Buhring L, Heydorn W, Nouraldeen A, Xiong W, Yalamanchili P (2014). Effect of LX4211 on glucose homeostasis and body composition in preclinical models. J Pharmacol Exp Ther.

[23] Bode D, Guthof T, Pieske BM, Heinzel FR, Hohendanner F (2018). Isolation of atrial cardiomyocytes from a rat model of metabolic syndrome-related heart failure with preserved ejection fraction. J Vis Exp.

[24] Voigt N, Heijman J, Wang Q, Chiang DY, Li N, Karck M, Wehrens XHT, Nattel S, Dobrev D (2014). Cellular and molecular mechanisms of atrial arrhythmogenesis in patients with paroxysmal atrial fibrillation. Circulation.

[25] Hohendanner F, Walther S, Maxwell JT, Kettlewell S, Awad S, Smith GL, Lonchyna VA, Blatter LA (2015). Inositol-1,4,5-trisphosphate induced Ca2+ release and excitation-contraction coupling in atrial myocytes from normal and failing hearts. J Physiol.

[26] Hohendanner F, Ljubojevic S, MacQuaide N, Sacherer M, Sedej S, Biesmans L, Wakula P, Platzer D, Sokolow S, Herchuelz A (2013). Intracellular dyssynchrony of diastolic cytosolic [Ca(2)(+)] decay in ventricular cardiomyocytes in cardiac remodeling and human heart failure. Circ Res.

[27] Maxwell JT, Tsai CH, Mohiuddin TA, Kwong JQ (2018). Analyses of mitochondrial calcium influx in isolated mitochondria and cultured cells. J Vis Exp JoVE.

[28] Kolijn D, Pabel S, Tian Y, Lodi M, Herwig M, Carrizzo A, Zhazykbayeva S, Kovacs A, Fulop GA, Falcao-Pires I (2020). Empagliflozin improves endothelial and cardiomyocyte function in human heart failure with preserved ejection fraction via reduced pro-inflammatory-oxidative pathways and protein kinase Galpha oxidation. Cardiovasc Res.

[29] Hohendanner F, Maxwell JT, Blatter LA (2015). Cytosolic and nuclear calcium signaling in atrial myocytes: IP3-mediated calcium release and the role of mitochondria. Channels (Austin).

[30] Voigt N, Li N, Wang Q, Wang W, Trafford AW, Abu-Taha I, Sun Q, Wieland T, Ravens U, Nattel S (2012). Enhanced sarcoplasmic reticulum Ca2+ leak and increased Na+-Ca2+ exchanger function underlie delayed afterdepolarizations in patients with chronic atrial fibrillation. Circulation.

[31] Primessnig U, Bracic T, Levijoki J, Otsomaa L, Pollesello P, Falcke M, Pieske B, Heinzel FR (2019). Long-term effects of Na(+) /Ca(2+) exchanger inhibition with ORM-11035 improves cardiac function and remodelling without lowering blood pressure in a model of heart failure with preserved ejection fraction. Eur J Heart Fail.

[32] Uthman L, Baartscheer A, Bleijlevens B, Schumacher CA, Fiolet JWT, Koeman A, Jancev M, Hollmann MW, Weber NC, Coronel R (2018). Class effects of SGLT2 inhibitors in mouse cardiomyocytes and hearts: inhibition of Na(+)/H(+) exchanger, lowering of cytosolic Na(+) and vasodilation. Diabetologia.

[33] Maack C, Cortassa S, Aon MA, Ganesan AN, Liu T, O'Rourke B (2006). Elevated cytosolic Na+ decreases mitochondrial Ca2+ uptake during excitation-contraction coupling and impairs energetic adaptation in cardiac myocytes. Circ Res.

[34] Drago I, De Stefani D, Rizzuto R, Pozzan T (2012). Mitochondrial Ca2+ uptake contributes to buffering cytoplasmic Ca2+ peaks in cardiomyocytes. Proc Natl Acad Sci USA.

[35] Schweitzer MK, Wilting F, Sedej S, Dreizehnter L, Dupper NJ, Tian Q, Moretti A, My I, Kwon O, Priori SG (2017). Suppression of arrhythmia by enhancing mitochondrial Ca(2+) uptake in catecholaminergic ventricular tachycardia models. JACC Basic Transl Sci.

[36] Lemasters JJ, Theruvath TP, Zhong Z, Nieminen AL (2009). Mitochondrial calcium and the permeability transition in cell death. Biochem Biophys Acta.

[37] Brown DA, O'Rourke B (2010). Cardiac mitochondria and arrhythmias. Cardiovasc Res.

[38] Lindman BR, Davila-Roman VG, Mann DL, McNulty S, Semigran MJ, Lewis GD, de las Fuentes L, Joseph SM, Vader J, Hernandez AF (2014). Cardiovascular phenotype in HFpEF patients with or without diabetes: a RELAX trial ancillary study. J Am Coll Cardiol.

[39] Cefalo CMA, Cinti F, Moffa S, Impronta F, Sorice GP, Mezza T, Pontecorvi A, Giaccari A (2019). Sotagliflozin, the first dual SGLT inhibitor: current outlook and perspectives. Cardiovasc Diabetol.

[40] Tsimihodimos V, Filippas-Ntekouan S, Elisaf M (2018). SGLT1 inhibition: Pros and cons. Eur J Pharmacol.

[41] Connelly KA, Zhang Y, Desjardins JF, Thai K, Gilbert RE (2018). Dual inhibition of sodium-glucose linked cotransporters 1 and 2 exacerbates cardiac dysfunction following experimental myocardial infarction. Cardiovasc Diabetol.

[42] Li Z, Agrawal V, Ramratnam M, Sharma RK, D'Auria S, Sincoular A, Jakubiak M, Music ML, Kutschke WJ, Huang XN (2019). Cardiac sodium-dependent glucose cotransporter 1 is a novel mediator of ischaemia/reperfusion injury. Cardiovasc Res.

[43] Yoshii A, Nagoshi T, Kashiwagi Y, Kimura H, Tanaka Y, Oi Y, Ito K, Yoshino T, Tanaka TD, Yoshimura M (2019). Cardiac ischemia-reperfusion injury under insulin-resistant conditions: SGLT1 but not SGLT2 plays a compensatory protective role in diet-induced obesity. Cardiovasc Diabetol.

[44] Leong HS, Grist M, Parsons H, Wambolt RB, Lopaschuk GD, Brownsey R, Allard MF (2002). Accelerated rates of glycolysis in the hypertrophied heart: are they a methodological artifact?. Am J Physiol Endocrinol Metab.

[45] Piao L, Sidhu VK, Fang YH, Ryan JJ, Parikh KS, Hong Z, Toth PT, Morrow E, Kutty S, Lopaschuk GD (2013). FOXO1-mediated upregulation of pyruvate dehydrogenase kinase-4 (PDK4) decreases glucose oxidation and impairs right ventricular function in pulmonary hypertension: therapeutic benefits of dichloroacetate. J Mol Med (Berl).

[46] Fillmore N, Levasseur JL, Fukushima A, Wagg CS, Wang W, Dyck JRB, Lopaschuk GD (2018). Uncoupling of glycolysis from glucose oxidation accompanies the development of heart failure with preserved ejection fraction. Mol Med.

[47] Kohlhaas M, Nickel AG, Maack C (2017). Mitochondrial energetics and calcium coupling in the heart. J Physiol.

[48] Shao Q, Meng L, Lee S, Tse G, Gong M, Zhang Z, Zhao J, Zhao Y, Li G, Liu T (2019). Empagliflozin, a sodium glucose co-transporter-2 inhibitor, alleviates atrial remodeling and improves mitochondrial function in high-fat diet/streptozotocin-induced diabetic rats. Cardiovasc Diabetol.

