# Supplementary material for: Dual SGLT-1 and SGLT-2 inhibition improves left atrial dysfunction in HFpEF
Source: Cardiovasc Diabetol. 2021 Jan 7;20:7. doi: 10.1186/s12933-020-01208-z (PMC7792219; doi:10.1186/s12933-020-01208-z)
Supplement: Supplementary file 1 — Additional file 1: Figure S1. (A) Longitudinal and (B) transversal LV fractional shortening as observed in echocardiography (parasternal long axis view). (C) Correlation of longitudinal and (D) transversal LV fractional shortening with LA volume. (E) Exemplary electrocardiogram to monitor atrial fibrillation (excerpt of 30 min continuous recording). (F) Total occurrence of atrial fibrillation during screened time intervals. (G) Serum concentration of creatinine and (H) urea. Statistical analysis: Two-way ANOVA followed by post-hoc Bonferroni (G, H). n = animals. 10.016, 2 < 0.001, 3 < 0.001, 40.014, 5 < 0.001. Figure S2. (A) Correlation of diastolic Ca2+, (B) CaT amplitudes, (C) CaT time-to-peak (TTP), (D) CaT tau of decay, (E) diastolic sarcomere length, (F) sarcomere shortening, (G) sarcomere time-to-peak (TTP) and sarcomere RT50 (H) of LV and LA cardiomyocytes. p-values (deviation from zero): 1 < 0.0001, 20.005, 30.01. n = average per animal. Figure S3. (A) Western Blot of LA SGLT-1 expression (above) and respective total protein visualized by Ponceau S staining (below). (B) Corresponding statistical analysis of SGLT-1 signal intensity after normalization to total protein. (C) Western Blot of LA SGLT-2 expression (above). and respective total protein (below), kidney (K) shown as positive control. (D) Corresponding SGLT-2 signal intensity after normalization to total protein. Statistical analysis: Two-way ANOVA followed by Fisher’s LSD test. n = animals. 10.003, 20.048, 30.03. Figure S4. (A) Example of polarized mitochondria (TMRE / MitoTracker binary overlay after local thresholding) at baseline (left) and after spontaneous depolarization (right) and corresponding signal trace (below). (B) Standard deviation (SD) of signal trace over the course of 6 min. (C) Maximum depolarization per cell. Statistical analysis: Two-way ANOVA followed by post-hoc Bonferroni. Figure S5. (A) Example of SCaEs during a non-stimulated interval, following 3 Hz stimulation for 10 s at b [file 12933_2020_1208_MOESM1_ESM.docx]

Figure S1


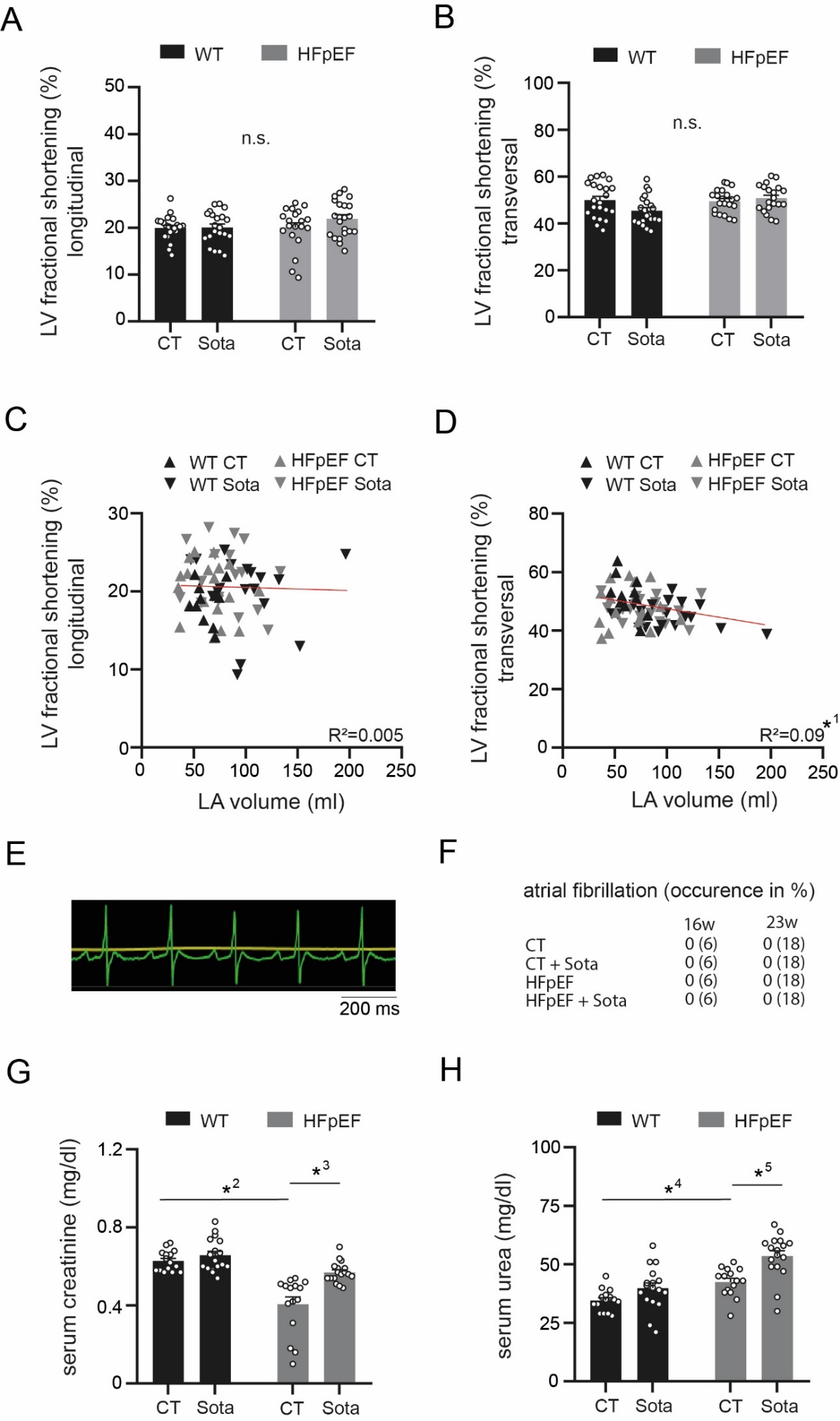


Figure S1. (A) Longitudinal and (B) transversal LV fractional shortening as observed in echocardiography (parasternal long axis view). (C) Correlation of longitudinal and (D) transversal LV fractional shortening with LA volume. (E) Exemplary electrocardiogram to monitor atrial fibrillation (excerpt of 30 min continuous recording). (F) Total occurrence of atrial fibrillation during screened time intervals. (G) Serum concentration of creatinine and (H) urea. Statistical analysis: Two-way ANOVA followed by post-hoc Bonferroni (G, H). n = animals. ^1^0.016, ^2^<0.001, ^3^<0.001, ^4^0.014, ^5^<0.001.

Figure S2
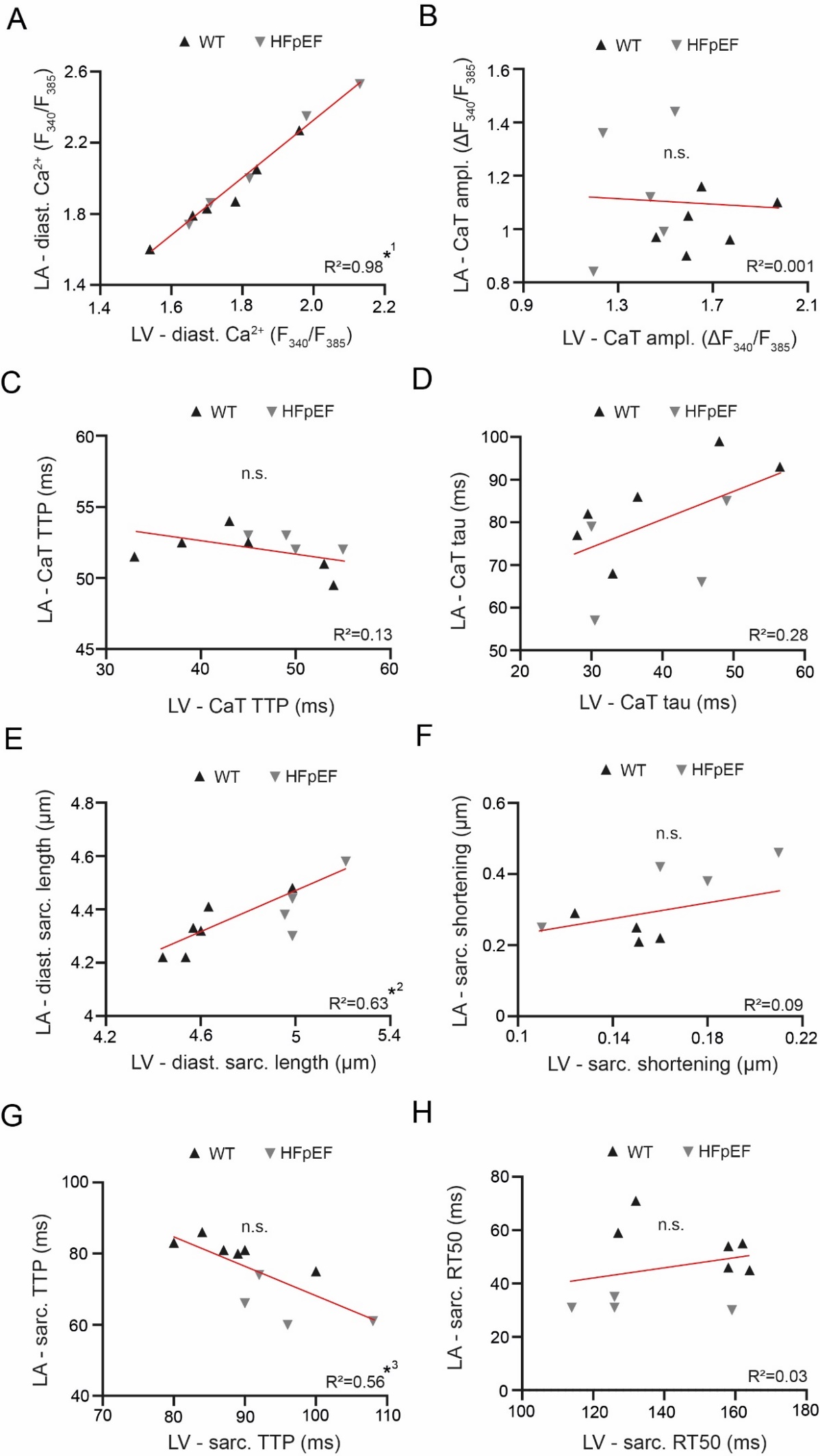


Figure S2. (A) Correlation of diastolic Ca^2+^, (B) CaT amplitudes , (C) CaT time-to-peak (TTP), (D) CaT tau of decay, (E) diastolic sarcomere length, (F) sarcomere shortening, (G) sarcomere time-to-peak (TTP) and sarcomere RT50 (H) of LV and LA cardiomyocytes. *p*-values (deviation from zero): ^1^<0.0001, ^2^0.005, ^3^0.01. n = average per animal.

Figure S3


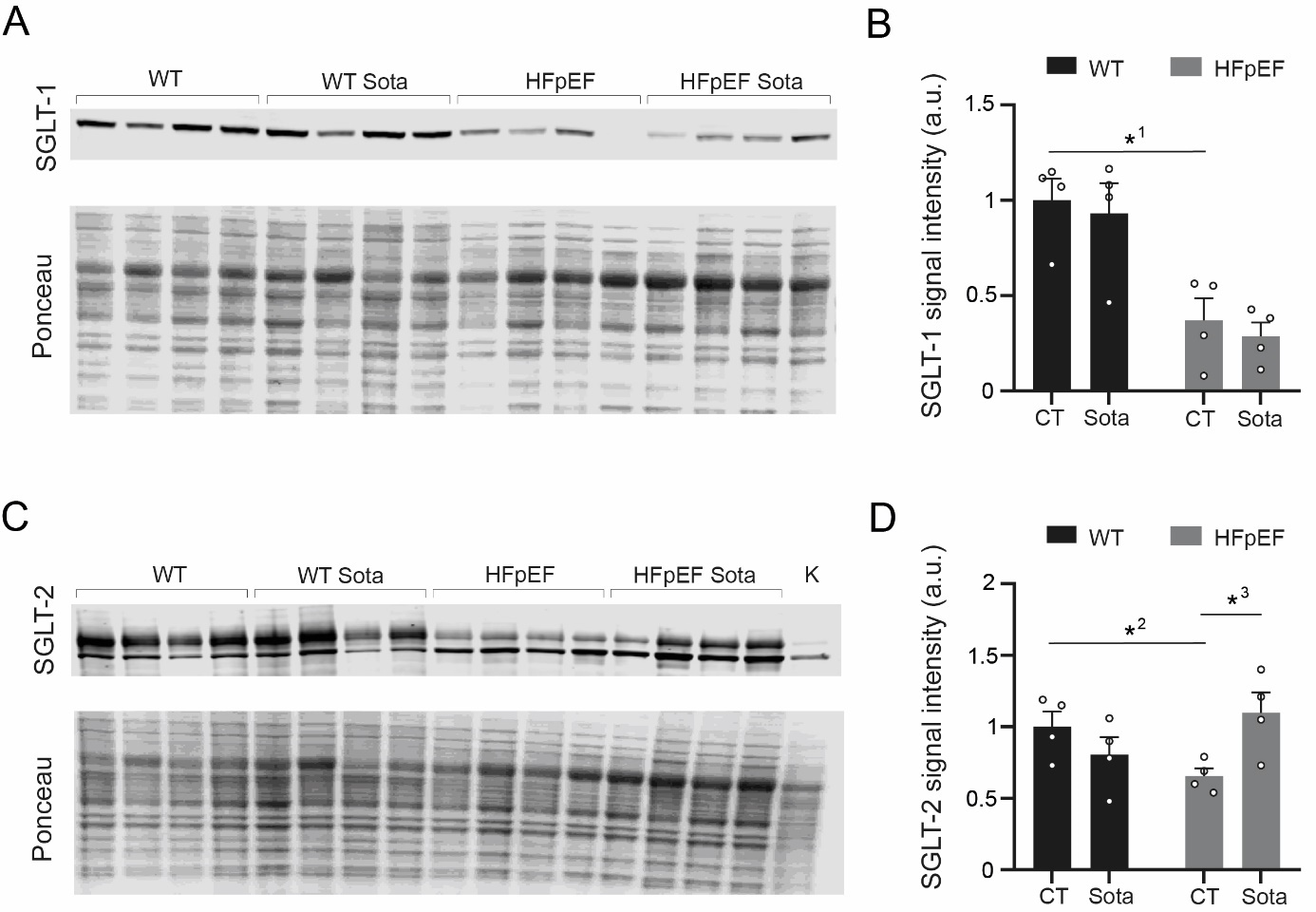


(A) Western Blot of LA SGLT-1 expression (above) and respective total protein visualized by Ponceau S staining (below). (B) Corresponding statistical analysis of SGLT-1 signal intensity after normalization to total protein. (C) Western Blot of LA SGLT-2 expression (above). and respective total protein (below) , kidney (K) shown as positive control. (D) Corresponding SGLT-2 signal intensity after normalization to total protein. Statistical analysis: Two-way ANOVA followed by Fisher’s LSD test. n = animals. ^1^0.003, ^2^0.048, ^3^0.03.

Figure S4


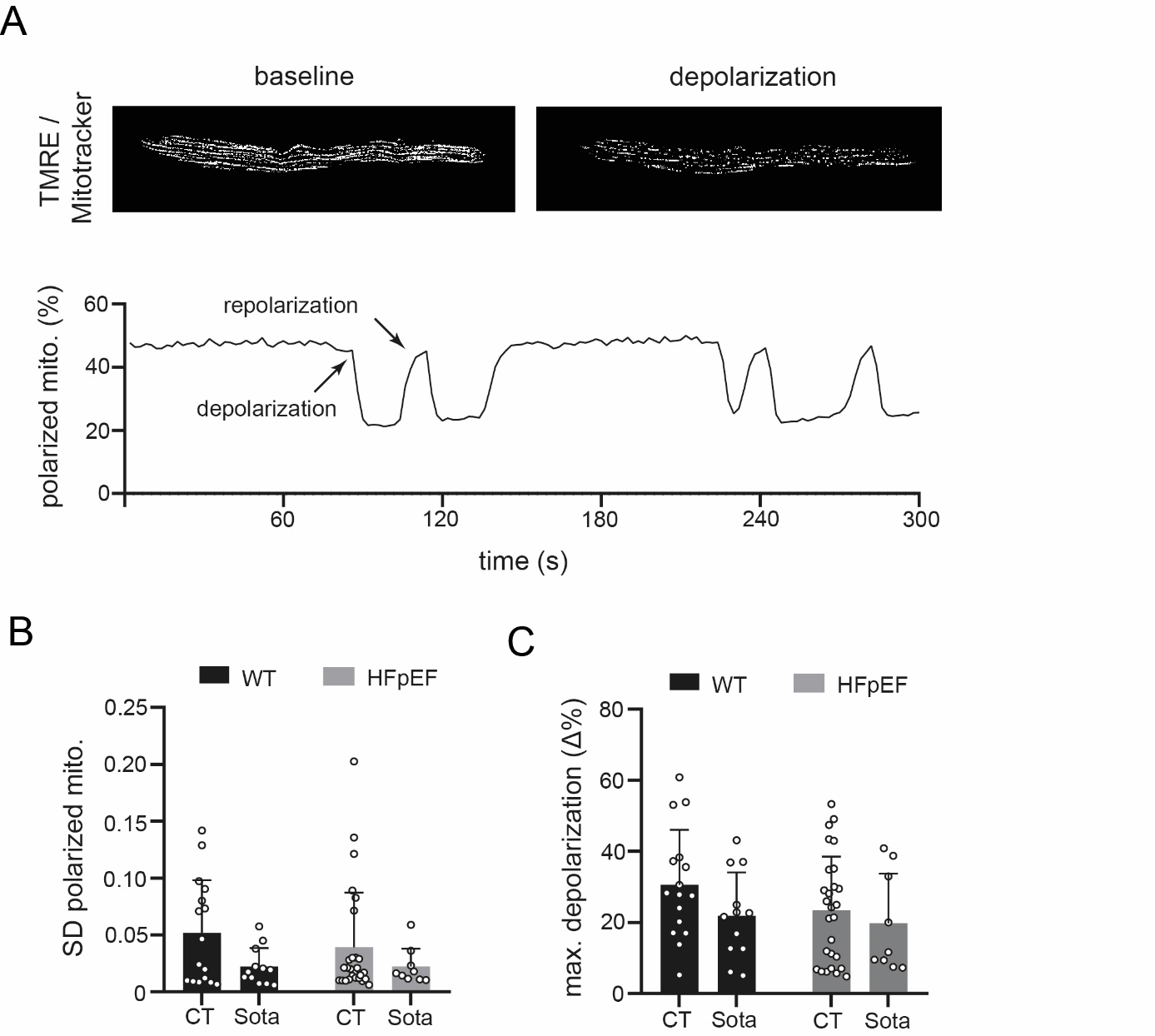


(A) Example of polarized mitochondria (TMRE / MitoTracker binary overlay after local thresholding) at baseline (left) and after spontaneous depolarization (right) and corresponding signal trace (below). (B) Standard deviation (SD) of signal trace over the course of 6 min. (C) Maximum depolarization per cell. Statistical analysis: Two-way ANOVA followed by post-hoc Bonferroni.

Figure S5


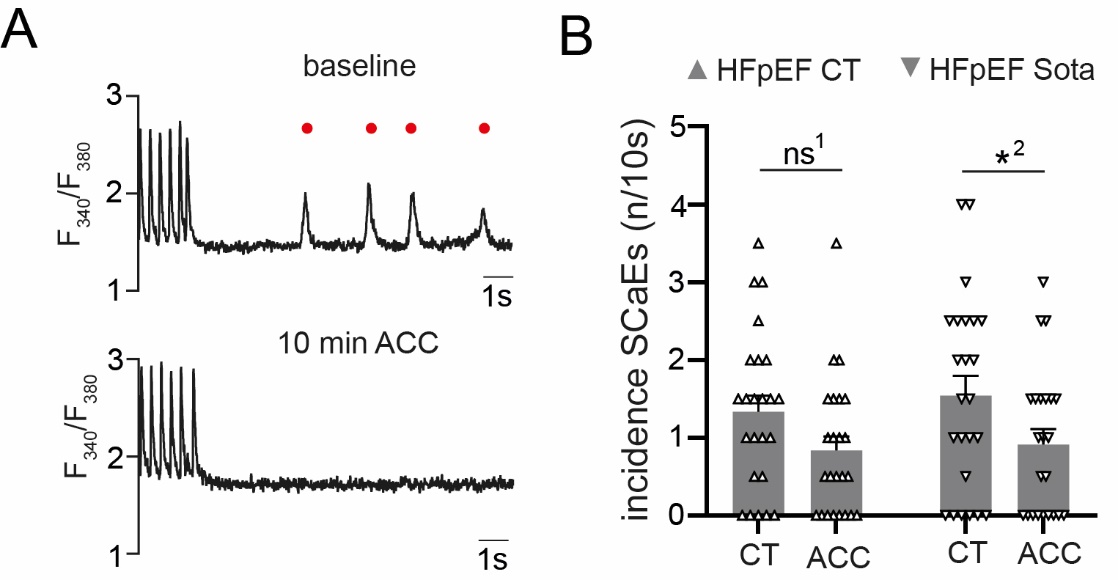


(A) Example of SCaEs during a non-stimulated interval, following 3 Hz stimulation for 10 s at baseline (above) and after 10 min incubation with acetylcysteine (ACC; below). (B) Related occurrence of SCaEs (all HFpEF). Statistical analysis: Two-way ANOVA followed by post-hoc Bonferroni. *p*-values: ^1^0.069, ^2^0.018.
